# HOXA10 Expressing UCMSCs Transplantation Improved Endometrial Receptivity on Endometrial Injury

**DOI:** 10.2174/1574888X17666220919111814

**Published:** 2023-05-29

**Authors:** Meixian Wu, Yuanyuan Li, Yiwei Wang, Yifan Li, Jinghui Li, Jing Xie, Shuang Zhao, Lihua Sun

**Affiliations:** 1 Institute for Regenerative Medicine, Shanghai East Hospital, Frontier Science Center for Cellular Stemness & Cell Fate Editing, School of Life Sciences and Technology, Tongji University, Shanghai 200092, P.R. China;; 2 Department of Reproductive Medicine Center, Shanghai East Hospital, Tongji University, Shanghai 200123, P.R. China

**Keywords:** UCMSCs, HOXA10, lentivirus, endometrial injury, endometrial receptivity, embryo implantation

## Abstract

**Background:**

Endometrial injury is considered the major cause of female infertility. Traditional therapies such as estrogen substitution therapy are not satisfactory due to individual variation in response to treatment, thereby warranting the use of alternative strategies such as stem cell therapy. Transplantation of stem cells, such as umbilical cord mesenchymal stem cells (UCMSCs), has been shown to improve endometrial healing. However, due to the effect of the intrauterine environment, the therapeutic effect of UCMSCs is limited, and its efficacy is unstable. HOXA10, encoded by the *HOXA10* gene, plays an important role in endometrium morphology maintenance, proliferation, differentiation, and embryo implantation. Moreover, UCMSCs do not show HOXA10 expression.

**Objective:**

Our study aimed to evaluate the therapeutic effects of HOXA10-transfected UCMSCs on endometrial injury repair *in vivo*.

**Methods:**

First, we established T10-UCMSCs (UCMSCs transfected with HOXA10) for transplantation. To establish the endometrial injury model, we injected 95% ethanol into the uterine cavity and transplanted T10-UCMSCs into the uterine cavity from the cornua uteri. Fourteen days later, uteri were collected for histological and biochemical analysis of endometrial growth and receptivity.

**Results:**

Our results showed the endometrial receptivity was better in T10-UCMSCs group than in UCMSCs group, suggesting that HOXA10 could enhance the repairing ability of UCMSCs in the endometrium injury repair. More importantly, the fertility test showed that more embryos were implanted in the T10-UCMSCs group.

**Conclusion:**

Our results suggest that UCMSCs with HOXA10 expression could improve the therapeutic effects on endometrial injury repairing.

## 1 INTRODUCTION

Infertility is common among childbearing couples, with the rate of infertility rising every year, ranging between 8.5–20% in developed countries and 12–15% in China [1]. According to the World Health Organization (WHO), infertility is projected to be the third major disease following cancer and cardiovascular diseases [2]. Although people are seeking help for assisted reproductive technology (ART), problems associated with the endometrium limit the outcome of ART and cause female reproductive dysfunction [3]. Due to the complex pathogenesis of female reproductive dysfunction, effective clinical treatment is still lacking.

The endometrium is a highly dynamic tissue with regenerative ability. Anatomically, it consists of a functional layer and basal layer. The basal layer is relatively stable, while the functional layer is dynamic. The functional layer accounts for 2/3 of the endometrium, which undergoes a cycle of desquamation and regeneration under the regulation of ovarian hormones, especially estrogen and progesterone [4, 5]. However, an increasing number of women are suffering from an endometrial injury due to intrauterine surgery, inflammation, and curettage. Thin endometrium (TE, endometrium thickness<7 mm in the middle luteal phase) and intrauterine adhesion (IUA, a symptom of damage in the basal layer, scar formation, and failure of endometrial repair) are caused by endometrial injury [6, 7], and both of them can lead to embryo implantation failure and repeated miscarriages [8, 9]. Some traditional therapies, such as improving vascular perfusion and hormone replacement in clinical trials, may promote the regeneration of the endometrium to some extent [10-12]. However, the curative effect is not satisfactory due to individual variation.

Stem cell therapy is becoming increasingly promising in regenerative medicine. Several studies have shown that stem cells have regenerative effects, such as accelerating cartilage repair and improving ischemic organ recovery [13-15]. Moreover, several clinical trials are underway to treat endometrial diseases with stem cells from different sources. Among those, UCMSCs are considered promising seed cells due to their low immunogenicity,extensive source and lack of ethical dispute. Researchers discovered that injecting UCMSCs intravenously into mice boosted endometrial thickness and alleviate the fibrosis [16, 17]. However, functional indicators such as the rate of embryo implantation remain unstable.

*HOXA10*, a member of the Hox gene family, is mainly expressed in the endometrium and plays an important role in endometrial maintenance and embryo implantation [18, 19]. Female homozygotes with *HOXA10* deficiency died between days 2.5 and 3.5 post coitum [20]. Blocking of maternal *HOXA10* by antisense oligonucleotide inhibited embryo implantation, while transgenic mice with *HOXA10* overexpression had a higher litter size than wild-type mice [21, 22]. Previous studies have shown that the HOXA10 expression levels decreased significantly after endometrial injury repair while being upgraded after treatment [23]. Moreover, abnormal decrease of HOXA10 expression can lead to embryo implantation failure in ART treatment in the clinic [24]. Therefore, it remains unknown whether stem cells with HOXA10 expression can enhance endometrial injury repair and improve endometrial receptivity. To test this, we established a mouse endometrial injury model and transplanted HOXA10-expressing UCMSCs (T10-UCMSCs) to evaluate their therapeutic effect and endometrial function.

## 2 MATERIALS AND METHODS

### 2.1 Cell Culture

Human UCMSCs were isolated from umbilical cord tissues and identified by flow cytometry according to the standard process [25, 26]. The surface markers of UCMSCs were analyzed by flow cytometry with antibodies against CD11, CD19, CD31, CD34, CD45, CD73, CD90, CD105, and HLA-DR. Cells were cultured in DMEM/F12 media (Gibco, New York, USA) with 10% fetal bovine serum (FBS; Gibco, Shanghai, China) and 1% Penicillin-Streptomycin (Gibco, Shanghai, China) and incubated at 37 °C and 5% CO_2_ saturated humidity. After reaching 80% confluence, UCMSCs were digested with 0.25% trypsin (Gibco, New York, USA) for serial passage. UCMSCs or UCMSCs with HOXA10 expression (T10-UCMSCs) between passages 2 and 5 were used for transplantation.

### 2.2 Plasmid Preparation and Transfection

The sequence of *HOXA10* CDS was obtained from the NCBI database and cloned into a lentiviral vector (pLVX-AcGFP1-N1 plasmid). The lentiviral vector and lentiviral package plasmids (psPAX2 and pMD2.G) were co-transfected into HEK-293T cells, and the medium was replaced with a fresh medium after 12 h. After culturing for 48 h, the supernatant was filtered through a 0.45 μm syringe filter and mixed with 40% PEG 8000 at 4 °C overnight. Viruses were harvested by centrifuging at 3000 xG for 40 min, and stored at -80 °C. With the help of polybrene (Genomeditech, Shanghai, China), UCMSCs at 50% confluence were transfected with the virus. Seventy-two hours post-transduction, the viral genome was integrated into the host cell genome, and the transfection efficiency was approximately 74%.

### 2.3 Cell Proliferation Identified by Cell Counting Kit-8 (CCK8) Assay

After UCMSCs were infected by the lentivirus, we evaluated whether *HOXA10* affected cell proliferation by performing a CCK8 assay. Initially, we seeded 5 x 10^3^ cells into 96-well plates in each well and cultured them for 24 h with 100 μL/well. Ten microliters of CCK8 agent (HanBio, Shanghai, China) was added into the culture medium at 24, 48, 72, 96, and 120 h, respectively. Each set was cultured for 4 h, and the optical density at 450 nm was recorded using a microplate reader. The experiment was repeated for 3-5 wells in each set, and the testing values were averaged.

### 2.4 Dual-Luciferase Reporter Gene Assay

The *Emx2* or *ITGB3* promoter was inserted into a pGL3-basic vector (Promega, Beijing, China), respectively. Using Lipofectamine 2000 (Invitrogen, Waltham, USA), the promotor vector and *HOXA10* vector were co-transfected into HEK-293T cells as well as the pRL-SV40 plasmid. Forty-eight hours post-transfection, luciferase activity was detected by the dual-luciferase reporter assay system (Promega, Beijing, China) according to the manufacturer’s specifications, and the firefly luciferase activity was normalized to Renilla luciferase activity.

### 2.5 Endometrial Injury Model Establishment and Stem Cell Transplantation

To establish the mouse model of endometrial injury, mature female C57BL/6 mice between 6 to 10 weeks were raised under specific-pathogen-free (SPF) conditions in a temperature- and light-controlled room. The model was based on the rapid dehydration of ethanol according to the protocol described by Gao *et al*. [27]. After two consecutive estrous cycles, mice were anesthetized with 1% pentobarbital sodium in the third estrous cycle. The abdominal cavity was opened after mice lying in a prone position. The uteri were exposed and clamped with hemostatic clips. We injected 95% ethanol into the cornua uteri and stayed for 90 s. The ethanol was withdrawn and the uteri were gently flushed with large amounts of saline solution in case of abdominal adhesion. The identical procedure was performed on the other side of the uterus, which was then replaced and the abdominal cavity was closed. The sham group was treated with saline solution, while the other three groups were treated with 95% ethanol. One hundred mice were randomly divided into four groups: the sham group (injected saline solution into uteri during modeling, n=25), the surgery group (injected 95% ethanol into uteri for modeling, n=25), the UCMSCs group (injected UCMSCs into uteri after modeling, n= 25), and the T10-UCMSCs group (injected T10-UCMSCs into uteri after modeling, n = 25).

Except for the sham group and the surgery group, the other two groups were transplanted with UCMSCs and T10-UCMSCs cell suspension, respectively. The digested stem cells were prepared in advance and were counted before transplantation. The operation was referred to as the model establishment process. The cell suspension was inserted with insulin syringes, and it was slowly injected from the cornua uteri, taking care of the overflow. The uteri were put back, and the abdominal cavity was closed after completing bilateral transplantation. Mice were placed on a temperature control table and were raised in cages until waking up.

### 2.6 Hematoxylin and Eosin Staining (HE staining)

After mice were sacrificed, uteri obtained were fixed with 4% paraformaldehyde for 24 h and embedded in paraffin. Tissue sections were deparaffinized in xylene (15 min twice), rehydrated in the decreasing ethanol series (100%, 100%, 95%, 85%, 75%, and 50%, 5 min each set), and washed with distilled water. The tissue sections were stained with hematoxylin for 10 min and rinsed in water. The tissue sections were differentiated with 1% hydrochloric acid alcohol for 30 s, rinsed in water, and finally stained with eosin for 2 min. After the color reaction, sections were dehydrated in an ethanol series and mounted by neutral gum. The vertical distance between the endometrial myometrial interface and the endometrial surface was measured as the thickness of the endometrium and was processed with image-processing software. The thickness and morphology of the endometrium in each group were evaluated.

### 2.7 Masson Staining

Following the instructions of the Masson-trichrome staining kit (Servicebio, Wuhang, China), paraffin slides were immersed into Masson A solution at room temperature overnight (approximately 15 h) and incubated at 65 °C for 30 min. Then, the sections were flushed with water for 30 s gently. The sections were immersed in a mixture of Masson B and Masson C for 1 min and flushed with water. Hydrochloric acid alcohol (1%) was used to differentiate the sections for 1 min until the nucleus appeared gray, while the background appeared colorless. The sections were drained and dip-dyed in Masson D for 6 min until a bright red color emerged. Masson E was applied to differentiate for 1 min, upon which the collagenous fibers appeared light red while fibers appeared red. After draining off the Masson E, the sections were immersed in Masson F for 2-30 s and rinsed with 1% glacial acetic acid three times in a row (8 s each time). The sections were dehydrated with ethanol three times in a row (5, 10, and 30 s each time). Finally, the sections were immersed in xylene twice (5 min each time).

### 2.8 Immunohistochemistry

The uteri were fixed in 4% paraformaldehyde for 24 h, embedded in paraffin, and sectioned at a thickness of 5 μm. After deparaffinization in xylene, sections were rehydrated in a decreasing ethanol series (100%, 100%, 95%, 85%, 75%, and 50%, 5 min each set) and rinsed in distilled water. Then, the sections were treated with 0.3% H_2_O_2_ for 15 min at room temperature to quench endogenous peroxidase activity. The sections were immersed in citrate buffer at 80 °C for 10 min for antigen retrieval. 10% normal goat serum (diluted with PBS for 1 h at room temperature was used for blocking and then incubated with primary polyclonal antibodies against vimentin (ab92547, Abcam) and VEGF (ab1316, Abcam) overnight at 4 °C. After being washed, the sections were incubated with secondary antibody diluted in 10% goat serum (1:1000) for 2 h.

### 2.9 Reverse Transcription and Quantitative Polymerase Chain Reaction Analysis

Total RNA was extracted by TRIzol reagent (Invitrogen, Carlsbad, USA), and cDNA was synthesized using a reverse transcription kit (Takara, Beijing, China) according to the manufacturer’s protocol. To normalize the expression of target genes, GAPDH was used as an internal control. We made a table list showing the primer sequences, product sizes, and annealing temperatures (Table **1**). Quantitative polymerase chain reaction (qRT-PCR) was performed using the Applied Biosystems 7500 Fast Real-Time PCR Detection System (Applied Biosystems, Shanghai, China), and each gene was amplified in triplicate. The reaction mixture of a 20 μL final volume contained10 μL of SYBR Green PCR Master Mix (Takara, Beijing, China), 2 μL of cDNA, 1 μL of forward and reverse primers, respectively, and ddH_2_O. PCR conditions were as follows: denaturation at 95 °C for 5 min, followed by 35 cycles at 94 °C for 45 s, 60 °C for 45 s, and 72 °C for 5 s, and extension at 72 °C for 10 min.

### 2.10 Western Blot

Mice uteri were homogenized in RIPA lysis buffer and centrifuged at 10000 xG for 15 min at 4 °C to transfer the supernatant. Protein samples were separated by sodium dodecyl sulfate-polyacrylamide gel electrophoresis after measuring their concentrations and were transferred to a polyvinylidene fluoride membrane. The membrane was blocked with Tris-buffered saline (TBS) containing 5% BSA for 1 h and incubated with primary antibodies overnight at 4 °C. The primary antibodies used were as follows: HOXA10 (ab191470, Abcam), cytokeratin (sc-70926, Santa Cruz), ITGB3 (ab179473, Abcam), LIF (ab138002, Abcam), GAPDH (HRP-60004, Protintech). After washing in TBS three times, membranes were incubated with 1:1000 peroxidase-conjugated goat anti-rabbit or mouse immunoglobulin G (IgG) for 2 h at room temperature.

### 2.11 Fertility Test

A total of thirty-seven mice were divided into four groups, including the sham group (n=7), the surgery group (n=9), the UCMSCs group (n=11), and the T10-UCMSCs group (n=10). Fourteen days after transplantation, female mice were mated with male mice in the same period at 1:1 ratio, and the same treatment was applied to the sham and surgery groups. Once vaginal plugs appeared, female mice were separated into another cage, and others were allowed to mate for a week until vaginal plugs were presented. Mice were sacrificed 17 days after plug appearance, and embryo implantation was observed in the uterus.

### 2.12 Statistical Analysis

Data were presented as the mean ± standard deviation (SD), significant differences between two groups were examined by Student’s t-test, while those among more groups were evaluated by One-way ANOVA analysis was used for comparisons among different groups. Values with *p*<0.05 were considered statistically significant.

## 3 RESULTS

### 3.1 Establishment of T10-UCMSCs for Endometrial Injury Transplantation

**Fig. (1):**
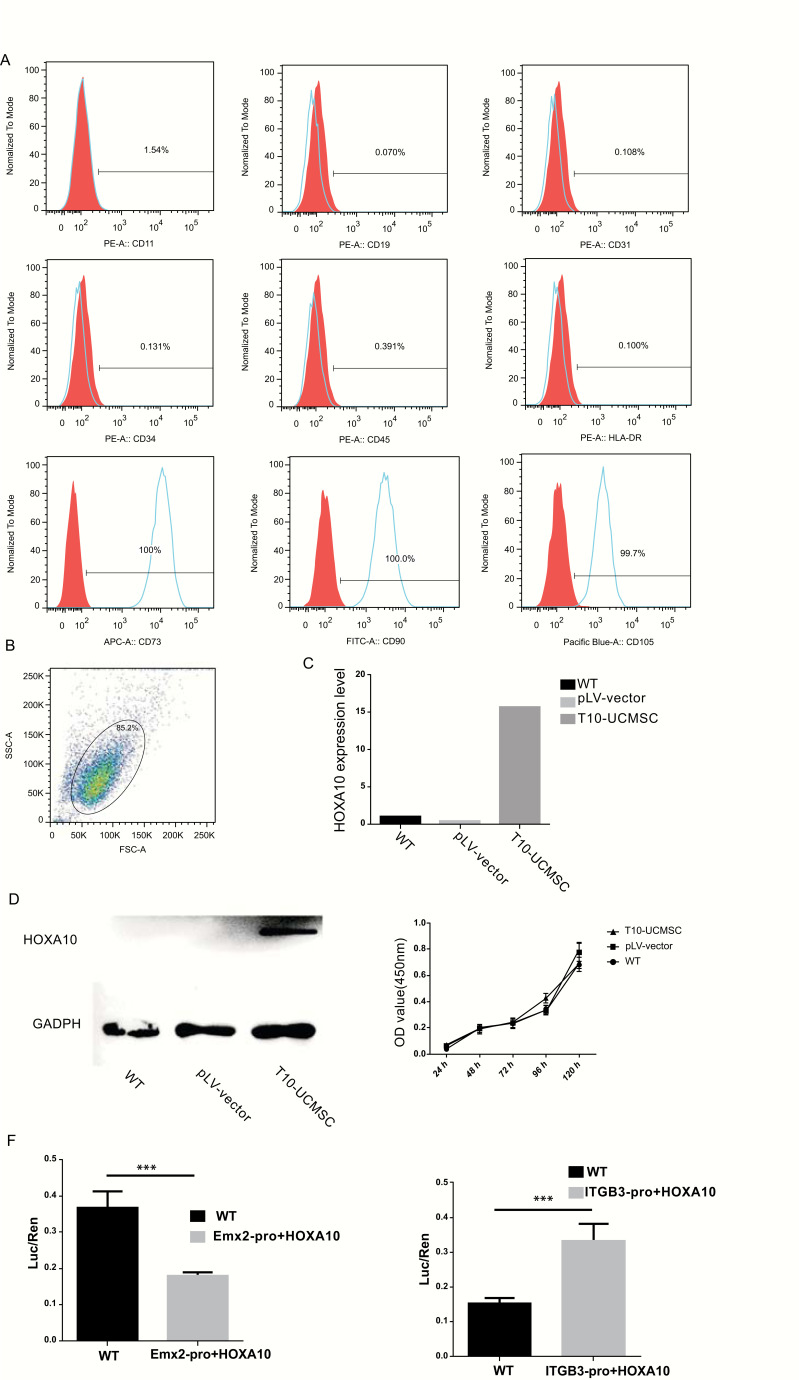
Establishment of T10-UCMSCs for endometrial injury transplantation. (**A**) The surface markers of umbilical cord mesenchymal stem cells (UCMSCs) (passage 2) were analyzed by flow cytometry. Antibodies against CD11, CD19, CD31, CD34, CD45, CD73, CD90 and CD105, and HLA-DR. (**B**) Percentage of positive cells in flow cytometry. (**C**, **D**) HOXA10 expression was confirmed by qRT-PCR and western blot analysis after lentiviral infection. (**E**) CCK8 assay of cell proliferation in the UCMSCs group, T10-UCMSCs, and UCMSCs groups with a lentiviral vector were used as controls. (**F**) The function of HOXA10 lentivirus was tested *in vivo* by dual-luciferase reporter gene assay, and the results demonstrated that HOXA10 exerted its effect by activating the downstream genes, *Emx2* and *ITGB3*. ***p<0.001.

As mentioned above, HOXA10 is involved in maintaining endometrial morphology, development, and receptivity. To evaluate the effects of HOXA10 on endometrial injury repair, we established a HOXA10-expressing UCMSCs cell line (T10-UCMSCs). First, the human UCMSCs were isolated and cultured. In passage 2, the surface markers of UCMSCs were analyzed by flow cytometry. The result showed that UCMSCs were negative for CD11 (1.54%), CD19 (0.070%), CD31 (0.108%), CD34 (0.131%), CD45 (0.391%), and HLA-DR (0.100%); while positive for CD73 (100%), CD90 (100%), and CD105 (99.7%) (Figs. **1A ** and **B **). After a rigorous surface biomarker screening, UCMSCs of high purity was obtained. HOXA10 was transfected into UCMSCs by lentiviral supernatant, and its expression was evaluated by qRT-PCR and western blot. Results of qRT-PCR showed *HOXA10* was highly expressed in UCMSCs after transfection (Fig. **1C**), which was consistent with western blot (Fig. **1D**). To test whether HOXA10 could affect the proliferation of UCMSCs, we performed the CCK8 assay to measure the cell growth, and no significant differences were observed between the T10-UCMSCs and the UCMSCs groups (Fig. **1E**). As a transcription factor, HOXA10 can suppress *Emx2* and activate *ITGB3* [28, 29]. To verify the functional activity of HOXA10, we performed a dual-luciferase reporter gene assay. Consistent with our expectation, the activity of the *Emx2* promoter decreased while that of the *ITGB3* promoter increased, suggesting that T10-UCMSCs were functionally ready for transplantation in mice (Fig. **1F**).

### 3.2 Endometrium Regeneration Was Promoted by Both UCMSCs and T10-UCMSCs

**Fig. (2):**
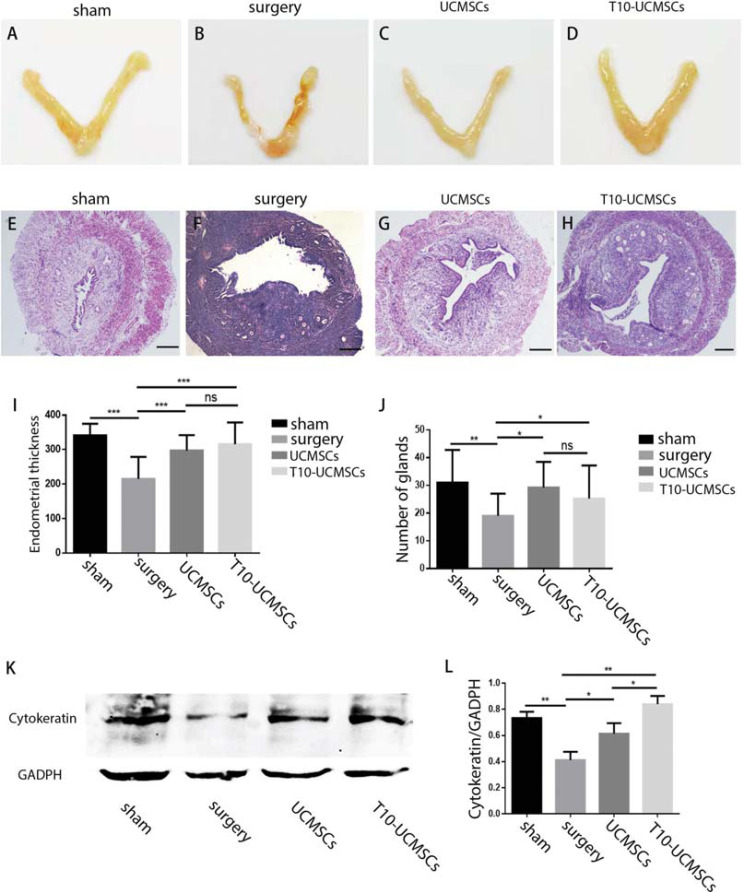
Endometrium regeneration was promoted by both UCMSCs and T10-UCMSCs. (**A-D**) The uteri shape of four groups (sham, surgery, UCMSCs, and T10-UCMSCs group). (**E**-**H**) The endometrial morphology in the four groups was examined by HE staining. 40x, scare bar = 150 μm. **I.** Statistic of endometrial thickness in (**E**-**H. J**) Statistic of glands of (**E**-**H. K**) The expression of cytokeratin in the endometrium was detected by western blotting. (**L**) Statistic of Y. ***p<0.001, **p<0.01, *p<0.5, no significance (ns)

To evaluate the therapeutic effects of T10-UCMSCs on endometrial injury, we established an endometrial injury model with 95% ethanol treatment. The T10-UCMSCs/UCMSCs were transplanted into the uterine cavity and fourteen days after transplantation, mice were executed, and uteri were collected for paraffin embedding. As shown in Figs. ( **2A** and **B**), the uterus was shrunken, and more blood stasis was observed in the surgery group. Notably, the symptoms were greatly relieved by stem cell transplantation (Figs. **2C ** and **D**). HE staining showed that the endometrium was not intact, and endometrial cells were sparse. The endometrial thickness was measured and quantified. The results showed that the endometrial thickness was higher in the stem cell transplantation groups than in the surgery group (sham group, 341.7±7.93 μm; UMCSC group, 297.4 ±12.93 μm, T10-UCMSCs group, 315.7±15.77 μm *vs.* surgery group, 215.5±15.46 μm) (Figs. **2E **-**I**). We counted the glands since endometrial damage reduced the number of glands. After transplantation, the number of glands increased significantly (UCMSCs group, T10-UCMSCs group *vs.* surgery group, *p*=0.0141 and *p*=0.0467, respectively) (Fig. **2J **). Furthermore, cytokeratin, which is expressed particularly in the cytoplasm of endometrial glandular or luminal epithelial cells [30], was considerably reduced after endometrial damage. Results of western blot showed that the level of cytokeratin was significantly decreased after injury (Figs. **2K ** and **L**, sham group *vs.* surgery group *p*=0.0022). The expression level of cytokeratin was slightly higher in the T10-UCMSCs group compared with that in the UCMSCs group (Figs. **2K ** and **L**, T10-UCMSCs group *vs.* UCMSCs group, *p*<0.5). However, the loss of cytokeratin after injury was not fully compensated. Taken together, both UCMSCs and T10-UCMSCs promoted the regeneration of the endometrium.

### 3.3 Endometrium Fibrosis Was Alleviated by Both UCMSCs and T10-UCMSCs

**Fig. (3):**
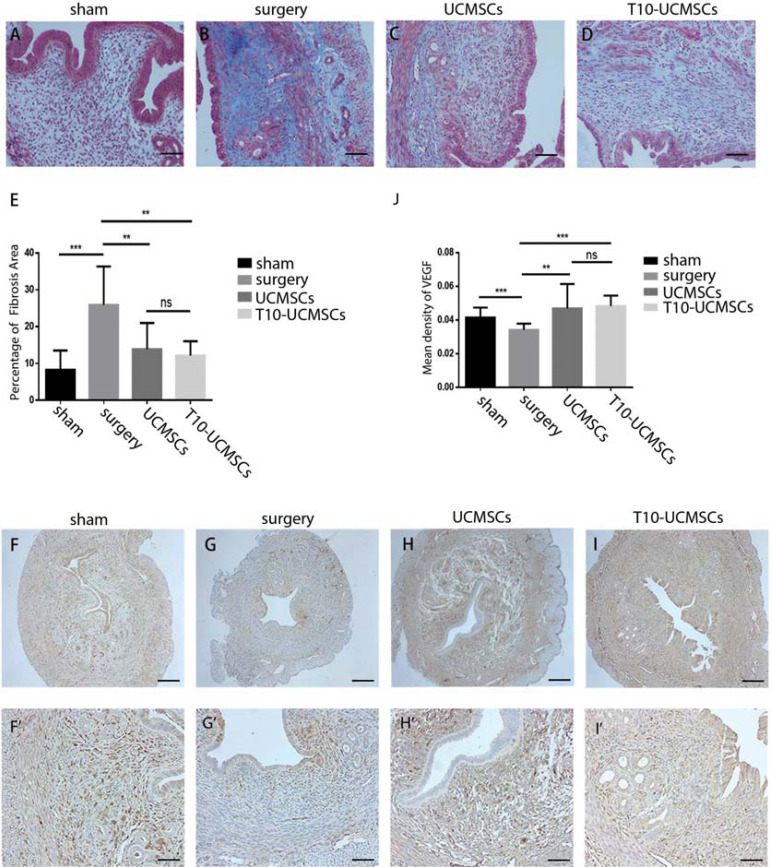
Endometrium fibrosis was alleviated by both UCMSCs and T10-UCMSCs. (**A**-**D**) The degree of fibrosis in four groups was examined by Masson staining. (**E**) Statistic of the fibrotic percentage area in (**A**-**D**). (**F**-**I**) Immunohistochemical staining of VEGF in four groups. F-I, 40x, scare bar = 150 μm, F’-I’, 100x, scare bar = 60 μm. (**J**) The percentage of VEGF-positive areas was analyzed in the endometrium of four groups. ***p<0.001, **p<0.01, *p<0.5, no significance (ns) UCMSCs and T10-UCMSCs groups seemed to have improved angiogenesis compared to the surgery group (Figs. **3H** and **I**, **H’** and **I’**, **3J**, UMCSCs group *vs*. surgery group, *p*=0.0019; T10-UCMSCs group *vs.* surgery group, *p*<0.0001).

Additionally, fibrosis is caused by the deposition of extracellular matrix, resulting in progressive tissue scarring and organ dysfunction [31]. Therefore, we performed Masson staining to evaluate the extent of fibrosis. As seen in Figs. ( **3A**- **E**), fibrosis was alleviated in the UCMSCs group (*p*=0.0141) and the T10-UCMSCs group (*p*=0.0467). Moreover, endometrial damage causes high uterine blood flow impedance and decreases the expression of vascular endothelial growth factor (VEGF), which plays an important role in endometrial angiogenesis [32]. Therefore, we performed immunohistochemistry to detect VEGF expression levels. The results showed that blood vessels were damaged and reduced in the surgery group (Figs. **3F**and **G**, **F’-G’**, **3J**; sham group *vs.* surgery group, *p*=0.0002), and both the

### 3.4 Endometrial Receptivity was Significantly Restored by T10-UCMSCs *vs.* UCMSCs

**Fig. (4):**
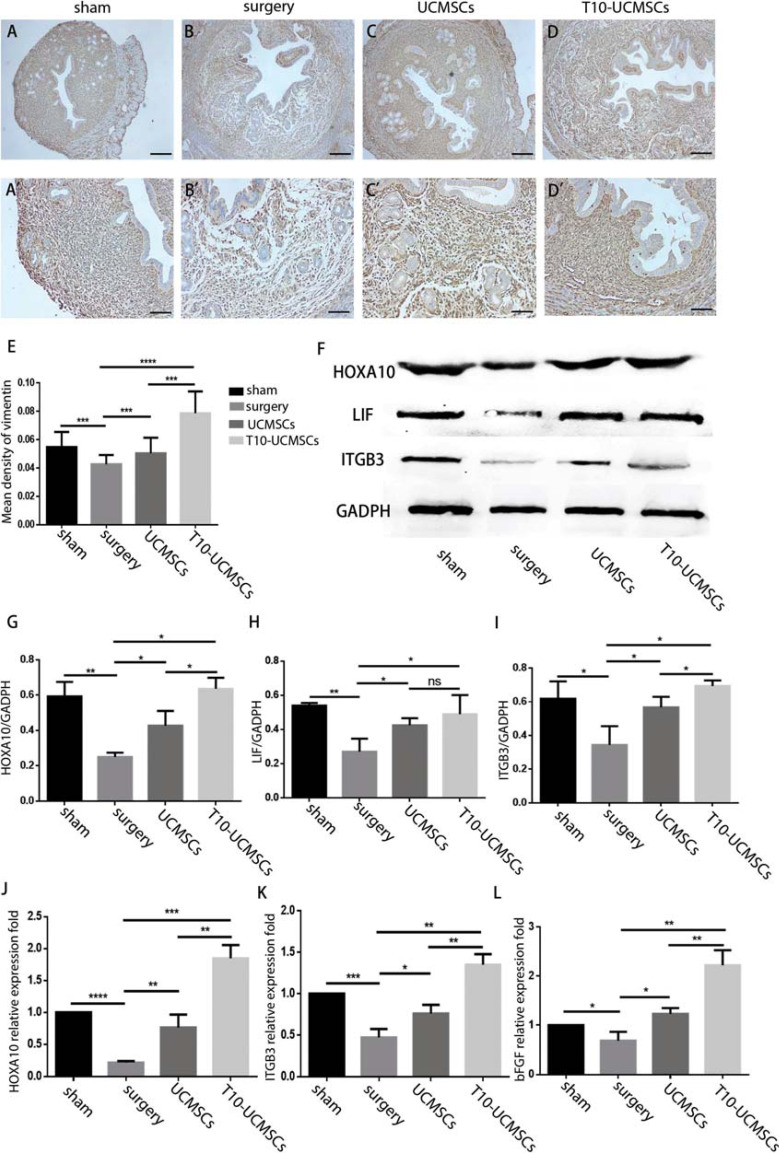
Endometrial receptivity was significant restored by T10-UCMSCs *vs.* UCMSCs. (**A-D**)’ Immunohistochemical staining of vimentin in four groups. (**A**-**D**), 40x scare bar = 150 μm, (**A**’-**D**)’, 100x scare bar = 60μm. (**E**) The percentage of VEGF-positive areas was analyzed in the endometrium of four groups. **F.** The expression of HOXA10, ITGB3, and LIF in the endometrium was detected by western blot. (**G**-**I**) Statistic of (**F**). (**J-L**) The expression of HOXA10, ITGB3, and bFGF in the endometrium was detected by qRT-PCR. ***p<0.001, **p<0.01, *p<0.5, no significance (ns)

The *HOXA10* gene is not only involved in endometrial development but also affects the decidualization of the endometrium as well as endometrial receptivity. Although there were no significant differences in the morphology between the T10-UCMSCs and UCMSCs groups, whether the endometrial receptivity was improved in the T10-UCMSCs group? To verify this, we evaluated the expression of endometrial receptivity biomarkers. Vimentin, specifically expressed in the endometrium interstitial cells, is considered an important endometrial receptivity biomarker [33]. Immunohistochemistry showed that the expression of vimentin in the T10-UCMSCs group was higher than that in the UCMSCs group (Figs. **4A **-**D**, **4A’**-**D’**, **4E **, sham group, UCMSCs group, T10-UCMSCs group *vs.* surgery group, *p*=0.0005, *p*=0.0205, *p*<0.0001, respectively; UCMSCs group *vs.* T10-UCMSCs group, *p*<0.001). Additionally, transplantation of UCMSCs and T10-UCMSCs resulted in an increase in the expression of HOXA10 (*p*=0.0242 and *p*=0.0006, respectively), ITGB3 (*p*=0.0390 and *p*=0.0065, respectively), and LIF (*p*=0.0457 and *p*=0.0037, respectively). Except for LIF, there was a significant difference in the expression of HOXA10 (*p*=0.0256) and ITGB3 (*p*=0.0368) between the UCMSCs and T10-UCMSCs groups (Figs. **4F **-**I**). Consistent with the western blot results, the qRT-PCR analysis showed a significantly higher expression of *HOXA10* (*p*=0.0097 and *p*=0.0002, respectively), *ITGB3* (p =0.0275 and *p*=0.0007 respectively), and *bFGF* (*p*=0.0457 and *p*=0.0037, respectively) in the UCMSCs and T10-UCMSCs groups compared to the surgery group (Figs. **4J **-**L**). All these results suggested that both UCMSCs and T10-UCMSCs improved endometrial receptivity in a HOXA10-dependent manner.

### 3.5 T10-UCMSCs Increased the Embryo Implantation Rate

**Fig. (5):**
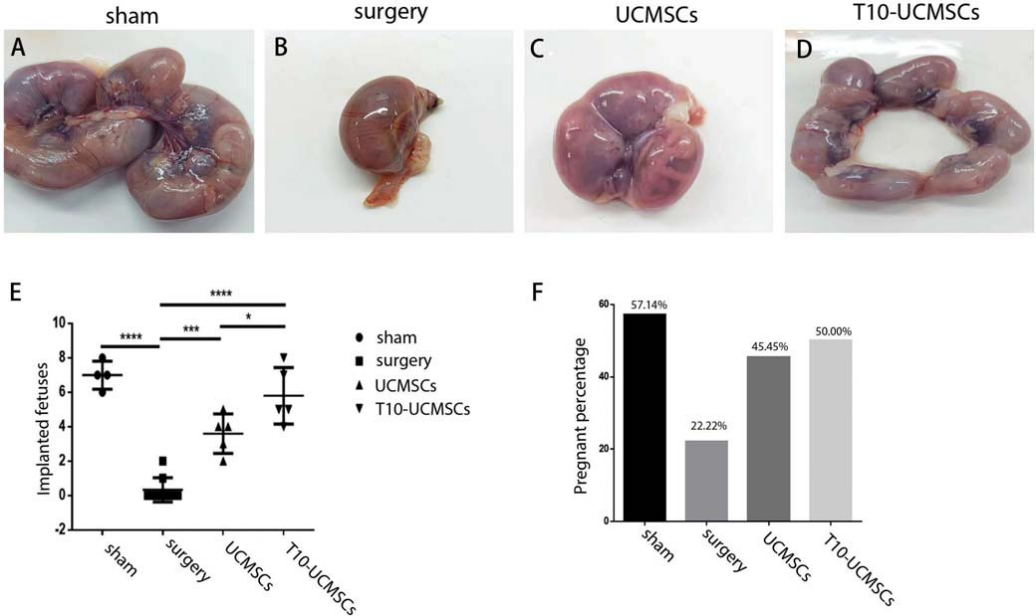
**UCMSCs increased the embryo implantation rate.** (**A**-**D**) Gross views of embryo implantation in sham, surgery, UCMSCs, and T10-UCMSCs groups. (**E**) Statistical analysis of the number of embryos in sham, surgery, UCMSCs, and T10-UCMSCs groups. (**F**) Percentage of pregnant mice in each group. ***p<0.001, **p<0.01, *p<0.5,.

Though the expression of endometrial receptivity markers was assessed, we further checked the implantation of embryos. To assess the effect of endometrial function repair, we performed a fertility test, which showed that both pregnancy and embryo implantation rates were increased in each group after mating at E17. There were only two pregnant mice in the surgery group (22.22% pregnancy rate), suggesting that endometrial injury caused infertility. Stem cell transplantation promoted the pregnancy rate with 45.45% in the UCMSC group and 50% in the T10-UCMSCs group (Fig. **5F**). Compared with the surgery group, the number of implanted embryos was significantly higher in the UCMSCs transplantation group (3.600 ± 0.5099, *p*<0.0001) and T10-UCMSCs transplantation group (5.800±0.7348, *p*<0.0001) (Figs. **5A-E **). The rate of implantation was higher in the T10-UCMSCs group than in the UCMSCs group (*p*=0.0393). The outcome of the fertility test indicated that both UCMSCs and T10-UCMSCs transplantation facilitated endometrial repair, and T10-UCMSCs transplantation had a better outcome in embryo implantation (Figs. **5A**-**F**).

## 4 DISCUSSION

Nowadays, intrauterine manipulation, drug abuse, and inflammation are major causes of endometrial injury and female infertility. Current therapies are aimed at regenerating the endometrium, although their effectiveness is limited. Meanwhile, stem cell therapy is associated with better outcomes in patients suffering from endometrial injury. Multiple studies have shown that UCMSCs can improve endometrial injury repair. For instance, UCMSCs transplantation restored the thickness of endometrium as well as the number of glands in an IUA rat model [34]. Moreover, in an intrauterine adhesion clinical treatment trial, combined with collagen scaffolds, UCMSCs could increase the endometrial thickness and relived intrauterine adhesion. Ten patients became pregnant after UCMSCs scaffolds transplantation [35]. Some positive outcomes have been achieved. However, due to individual variation and subsequent poor therapeutic efficiency, the results must be evaluated in a larger number of patients, with a particular focus on endometrial receptivity. HOXA10 is vital for the establishment of endometrial receptivity and embryo implantation [36]. As we mentioned previously, HOXA10 decreased in thin endometrium and increased after treatment [23]. *In vivo* blocking of maternal HOXA10 with antisense oligonucleotides resulted in reduced embryo implantation while transgenic mice overexpressed with HOXA10 had higher birth size [21, 22]. In the literature, there is no evidence of HOXA10 expression in UCMSCs, and the effect of HOXA10-expressing UCMSCs on endometrial injury repair remains unknown. In this study, we established a HOXA10-expressing UCMSC line (T10-UCMSCs) and evaluated its therapeutic effects on endometrial injury repair. Endometrial regeneration was observed in both wild-type UCMSCs and T10-UCMSCs transplantation, with an increased endometrial thickness, gland number, cytokeratin expression and decreased fibrosis and VEGF expression. Interestingly, although no significant changes in the endometrial morphology were observed between the UCMSCs- and T10-UCMSCs-transplanted group, endometrial receptivity and embryo implantation rate were significantly increased in the T10-UCMSCs-transplanted group. Since HOXA10 plays a major role in maintaining endometrial receptivity, these results suggest that T10-UCMSCs transplantation improves endometrial receptivity by increasing the expression of vimentin, HOXA10, and ITGB3.

Notably, the therapeutic mechanisms of UCMSCs transplantation are still being explored. Recently, multiple studies are trying to explore the mechanisms of UCMSCs in repairing endometrial injury. *In vitro*,UCMSCs have been proved effective in promoting the proliferation of HESC *via* paracrine effects, including VEGF-A, insulin-like growth factor 1, TGF- B and hepatocyte growth factor to inhibit cell apoptosis and promote angiogenesis [37]. Another study investigated the differential characteristics of the endometrial microenvironment including biological processes, molecular functions, cellular components, and pathways of endometrial injury and UCMSCs transplantation repair *via* miRNA and mRNA chip platforms. After UCMSCs transplantation, expression of 45 miRNAs was downregulated in the injured endometrium and upregulated and the expression of 39 miRNAs was upregulated in the injured endometrium and downregulated after UCMSCs transplantation [38]. Sun *et al*. found that miR-455-5p upregulation in umbilical cord mesenchymal stem cells attenuates endometrial injury and promotes repair of damaged endometrium *via* Janus kinase/signal transducer and activator of transcription 3 signaling [39]. Except for miRNAs, exosomes derived from

UCMSCs (UCMSCs-exos) have also become an important way of cell-free therapy recently. UCMSCs-exos could protect endometrial stromal cells(ESCs) from mifepristone-induced apoptosis and function in repairing the damaged ESCs by *in vitro* shuttling of miR-7162-3p, while APOL6 is involved in the regulation of apoptosis of ESC cells by UCMSC-exos-delivered miRNA [40]. The main limitation of our study is that the therapeutic mechanism remains unclear, and results showed most cytokines were upgraded after transplantation including cytokeratin, VEGF, vimentin and so on, indicating cell transplantation may function *via* paracrine effects. The therapeutic effects of stem cell transplantation are affected by transplantation methods [41]. Though intravenous transplantation is commonly used, homing of stem cells causes major loss, and only a few of them could remain at the damaged site. Endometrial injury is a type of uterine damage that can be treated by recruiting additional stem cells to the affected area [42]. Thus, we performed intrauterine transplantation directly to the damaged tissue to reduce the loss, and some repairing effects were observed. Multiple transplantations may be preferable in the future, such as intravenous transplantation as a supplement to intrauterine transplantation to increase the therapeutic effect. Moreover, stem cell residency at the injury site is another key factor in the therapeutic effect. Combined with bio-scaffold materials such as hyaluronic acid, collagen with stem cells has been considered a promising delivery strategy for stem cell therapy on endometrial regeneration [43]. We anticipate that great progress can be achieved in endometrial tissue engineering for clinical application with the new methods.

## CONCLUSION

Taken together, our study proved that HOXA10-expressing UCMSCs had better endometrial receptivity on endometrial injury repair, including morphology and function, and had an increased embryo implantation rate. In both animal models and clinical studies, we hope that a combination of better delivery systems and numerous transplantations will boost the therapeutic efficacy.

## Figures and Tables

**Table 1:** Primer sequence and product lengths for reverse transcription quantitative polymerase reaction.

**Gene**	**Primer Sequence**	**Product Lengths(bp)**
*HOXA10*	CCTGCCGCGAACTCCTTTT GGCGCTTCATTACGCTTGC	203
*ITGB3*	CCACACGAGGCGTGAACTC CTTCAGGTTACATCGGGGTGA	107
*bFGF*	GGAGAAGAGCGACCCACACG TGCCCAGTTCGTTTCAGTGC	270
*GAPDH*	TCCATGACAACTTTGGCATTG CAGTCTTCTGGGTGGCAGTGA	72

## Data Availability

Not applicable.

## LIST OF ABBREVIATIONS

GAPDH: Glyceraldehyde-3-Phosphate Dehydrogenase
HOXA10: Homeobox A10
UCMSCs: Human Umbilical Cord-Derived Mesenchymal Stem Cells
